# Efficacy of Colchicine for Secondary Prevention of Stroke: A Systematic Review and Meta-Analysis of Randomized Control Trials

**DOI:** 10.7759/cureus.75335

**Published:** 2024-12-08

**Authors:** Godfrey Tabowei, Hafiza Faiza Rauf, Milan Dhungana, Muhammad Awais, Keron Blair, Sandipkumar S Chaudhari, Ihtisham Habib, Adil Amin

**Affiliations:** 1 Department of Internal Medicine, Texas Tech University Health Sciences Center, Odessa, USA; 2 Medicine, Continental Medical College, Lahore, PAK; 3 Internal Medicine, Universal College of Medical Sciences, Bhairahawa, NPL; 4 Internal Medicine, Allama Iqbal Medical College, Lahore, PAK; 5 Medicine, American International School of Medicine, Georgetown, GUY; 6 Cardiothoracic Surgery, University of Alabama at Birmingham, Birmingham, USA; 7 Family Medicine, University of North Dakota School of Medicine and Health Sciences, Fargo, USA; 8 Internal Medicine, Medical Teaching Institute, Lady Reading Hospital Peshawar, Peshawar, PAK; 9 Cardiology, Pakistan Navy Station (PNS) Shifa, Karachi, PAK

**Keywords:** a systematic review and meta-analysis, colchicine, mortality, secondary prevention, stroke

## Abstract

Colchicine, a long-established anti-inflammatory medication, has emerged as a potential therapeutic agent for secondary prevention of stroke. This systematic review and meta-analysis aimed to evaluate the efficacy and safety of colchicine in preventing secondary stroke by comprehensively synthesizing available evidence. A systematic literature search was conducted across multiple electronic databases from inception to November 15, 2024, using comprehensive search strategies. Randomized controlled trials involving colchicine administration for stroke prevention were included. Two independent reviewers screened studies, extracted data, and assessed methodological quality using the Cochrane Risk of Bias tool. Meta-analysis was performed using Review Manager software, with risk ratios calculated for stroke incidence and all-cause mortality. The analysis encompassed seven studies involving 23,303 participants. The meta-analysis revealed a borderline significant 24% relative risk reduction in stroke incidence (risk ratio 0.76, 95% confidence interval 0.57-1.00, p = 0.05). Moderate heterogeneity was observed among studies (I² = 50%). Importantly, no significant difference was found in all-cause mortality between colchicine and control groups (risk ratio 1.03, 95% confidence interval 0.91-1.17, p = 0.66). While the findings suggest potential benefits of colchicine in stroke prevention, the results warrant cautious interpretation. The study emphasizes the need for larger, well-designed randomized controlled trials to definitively establish colchicine's role in comprehensive stroke prevention strategies.

## Introduction and background

Stroke is a major cause of morbidity and mortality globally, representing a significant burden on healthcare systems [1]. It is estimated that over 12 million people experience a stroke each year, with ischemic stroke accounting for approximately 87% of cases [2]. Despite advancements in the prevention and management of stroke, the recurrence rates remain high, particularly among patients with underlying atherosclerotic cardiovascular disease [3]. In this context, the identification of effective preventive strategies remains a key area of focus in reducing the global stroke burden. Chronic inflammation is increasingly recognized as a critical pathophysiological mechanism in the development and progression of atherosclerosis, the primary cause of ischemic stroke [4]. Inflammatory pathways play a central role in plaque formation, destabilization, and subsequent thrombosis, which can lead to cerebrovascular events [5]. Consequently, targeting inflammation has emerged as a promising strategy for stroke prevention, particularly in high-risk individuals with established cardiovascular disease [6]. 

Beyond its high mortality, stroke imposes a substantial burden on survivors and healthcare systems due to its severe and long-lasting impacts [7]. Stroke is a major cause of permanent disability, often resulting in hemiplegia, aphasia, and cognitive impairments that significantly affect the quality of life. Many patients are left with profound functional limitations, requiring long-term rehabilitation, caregiving, and support [8]. The economic impact of stroke is also considerable, driven by direct healthcare costs, lost productivity, and the need for ongoing care [9]. Given the rising prevalence and the severe consequences associated with stroke, particularly ischemic stroke, the focus on preventive measures has never been more critical [10]. 

Colchicine, a long-established anti-inflammatory medication traditionally used in the treatment of gout and familial Mediterranean fever, has gained attention for its potential cardiovascular benefits [11]. Colchicine exerts its anti-inflammatory effects by inhibiting microtubule assembly and reducing the activation of the NLRP3 inflammasome, a key mediator of the inflammatory response [12]. Recent clinical studies have highlighted the potential of colchicine to reduce the risk of major adverse cardiovascular events (MACE), including myocardial infarction and cardiovascular death, prompting interest in its potential role in preventing stroke [13]. 

Given the increasing interest in anti-inflammatory strategies for reducing stroke risk and the accumulating data on colchicine, a comprehensive synthesis of the available evidence is warranted. This systematic review and meta-analysis aim to evaluate the efficacy and safety of colchicine in the prevention of stroke. By pooling data from multiple studies, we aim to provide a more precise estimate of the potential benefits and risks associated with colchicine use, thereby informing clinical practice and guiding future research efforts in this area. 

## Review

Methodology 

A comprehensive literature search was conducted in multiple electronic databases, including PubMed, Embase, Cochrane Library, and Web of Science, from inception to November 15, 2024. The search strategy employed a combination of Medical Subject Headings (MeSH) terms and free-text keywords related to colchicine and stroke prevention. The main search terms included "colchicine," "stroke," "cerebrovascular accident," "brain ischemia," "prevention," and "prophylaxis." Additionally, reference lists of relevant articles and reviews were manually screened to identify any studies that might have been missed in the electronic search. No language restrictions were applied to ensure a thorough capture of all relevant literature. Search was conducted by two authors independently and any disagreement happens between them was resolved through discussion. 

Study Selection 

Two independent reviewers screened the titles and abstracts of all identified articles to assess their eligibility for inclusion. Studies were included if they met the following criteria: (1) randomized controlled trials with a control group; (2) adult participants (≥18 years old) (3) intervention involving colchicine administration for stroke prevention; and (4) reported outcomes including incidence of stroke and death. Studies were excluded if they were case reports, reviews, or animal studies. Full-text articles of potentially eligible studies were retrieved and independently assessed by the same two reviewers. Any disagreements were resolved through discussion or consultation with a third reviewer.

Data Extraction 

Data extraction was performed independently by two reviewers using a standardized, pre-piloted form. The extracted information included study characteristics (author, year of publication, study design, sample size), participant demographics, intervention details (dosage, duration of treatment), control group characteristics, outcome measures, and follow-up duration. For studies reporting multiple outcomes or time points, data for the longest follow-up period were extracted. Authors of included studies were contacted via email when clarification or additional data were required. Any discrepancies in data extraction were resolved through consensus or arbitration by a third reviewer. 

Quality Assessment 

The methodological quality of included randomized controlled trials was assessed using the Cochrane Risk of Bias tool 2.0, which evaluates bias across five domains: randomization process, deviations from intended interventions, missing outcome data, measurement of the outcome, and selection of the reported result. Two reviewers independently conducted the quality assessment, with any disagreements resolved through discussion or involvement of a third reviewer. 

Data Analysis 

Meta-analysis was performed using Review Manager (RevMan) version 5.4 software. The primary outcome was the incidence of stroke, and secondary outcomes included death. For dichotomous outcomes, risk ratios (RRs) with 95% confidence intervals (CIs) were calculated using a fixed-random or random-effects model based on level of heterogeneity. For outcomes where heterogeneity was less than 50%, fixed effect model was used. Otherwise, random-effect model was used to compute pooled estimates. P-value less than 0.05 was considered significant. Heterogeneity among studies was assessed using the I² statistic, with values of 25%, 50%, and 75% considered as low, moderate, and high heterogeneity, respectively. 

Results 

**Figure 1:**
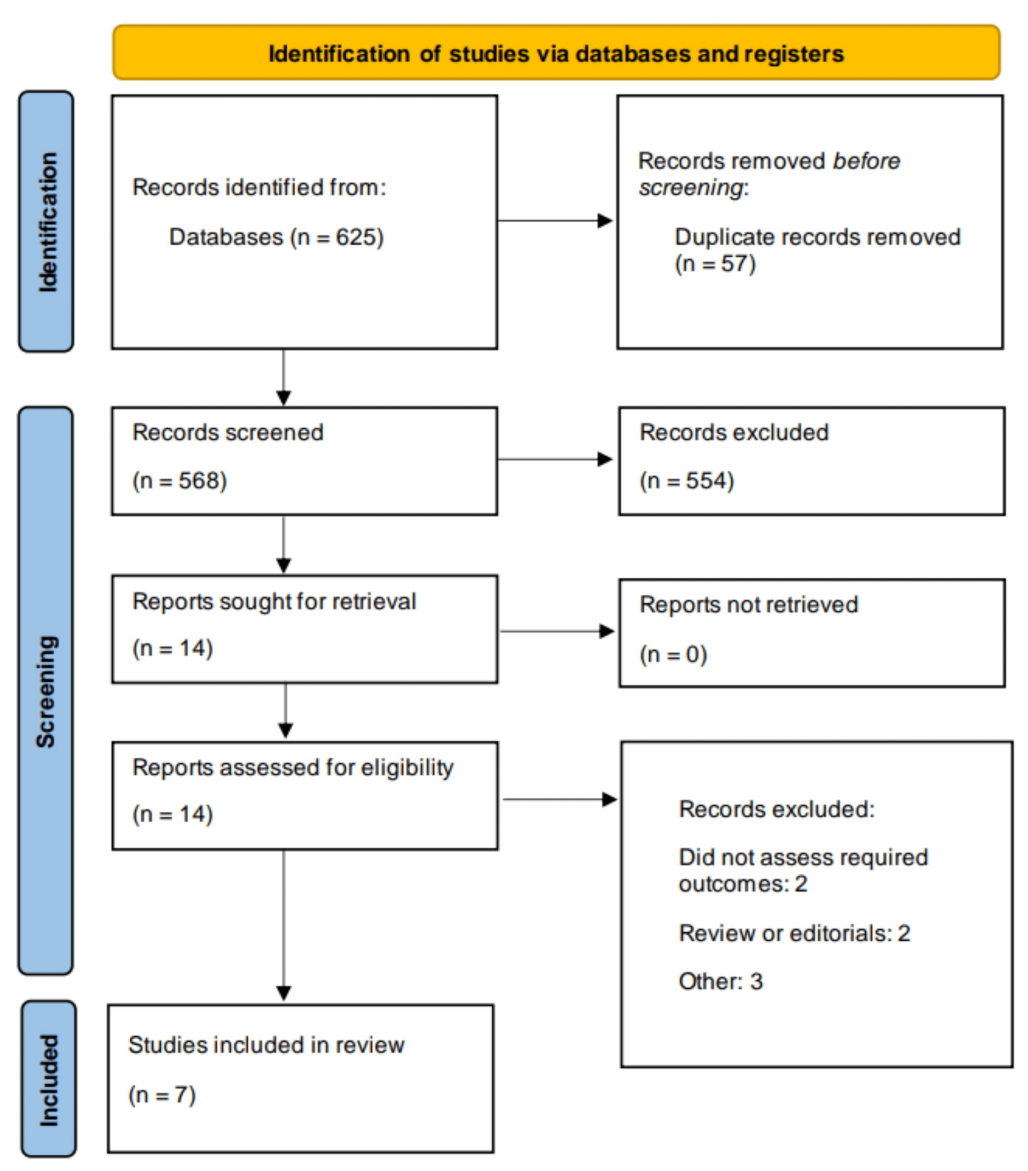
Preferred Reporting Items for Systematic Reviews and Meta-Analyses (PRISMA) flowchart

The systematic literature search yielded a total of 625 potentially relevant articles. After removing duplicates, 586 unique records were screened based on titles and abstracts. Of these, 14 full-text articles were assessed for eligibility, resulting in the inclusion of seven studies in the final analysis involving a total of 23303 participants. The sample sizes of individual studies ranged from 222 to 8343 participants. Figure 1 shows the Preferred Reporting Items for Systematic Reviews and Meta-Analyses (PRISMA) flowchart of study selection process. Table 1 presents the characteristics of all included studies. The duration of follow-up ranged from three months to 36 months. Colchicine dosages varied across studies, with the most common regimen being 0.5 mg once daily. 

**Table 1:** Characteristics of included studies (n= 7) NA: Not applicable; NR: Not reported

Author ID	Year	Trial Name	Setting	Groups	Sample Size	Follow-up	Dose	Age	Male	Hypertension	Diabetes
Deftereos [14]	2013	NA	Single Center	Colchicine	112	6 Months	Colchicine 0.5 mg twice daily	63.7	63	48	100
				Placebo	110			63.5	65	47	96
Kelly et al [15]	2024	CONVINCE	Multicenter	Colchicine	1569	36 Months	Colchicine 0.5 mg once daily	66.4	1081	1026	358
				Placebo	1575			66.2	1110	1031	343
Li et al [16]	2024	Chance 3	Multicenter	Colchicine	4176	3 Months	Colchicine 0.5 mg twice daily	66.3	2632	3175	1331
				Placebo	4167			66.4	2578	3223	1346
Nidorf [17]	2013	LoDoCo	Single Center	Colchicine	282	36 Months	Colchicine 0.5 mg once daily	66	251	NR	92
				Placebo	250			67	222		69
Nidorf [18]	2020	LoDoCo2	Multicenter	Colchicine	2762	28.6 Months	Colchicine 0.5 mg once daily	65.8	2305	1421	632
				Placebo	2760			65.9	2371	1387	662
Tardif [19]	2020	COLCOT	Multicenter	Colchicine	2366	22.6 Months	Colchicine 0.5 mg twice daily for one month, followed by 0.5 mg once daily	60.6	1894	1185	462
				Placebo	2379			60.5	1942	1236	497
Tong et al [20]	2021	COPS	Multicenter	Colchicine	396	12 Months	Colchicine 0.5 mg once daily	59.7	322	201	75
				Placebo	399			60	310	199	76

Effect of Colchicine on Risk of Stroke 

**Figure 2:**
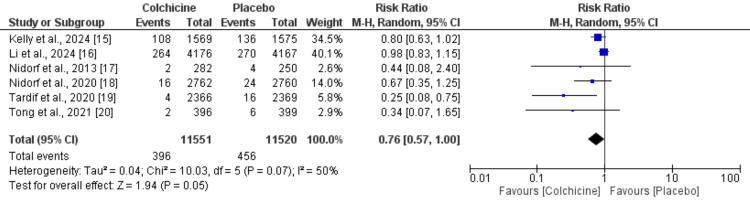
Effect of colchicine on stroke References [15-20]

A total of six studies were included in the meta-analysis, comparing the effect of colchicine on stroke prevention against control groups and the results are shown in Figure 2. The pooled analysis involved 11,551 participants in the experimental (colchicine) group and 11,520 participants in the control group. Across the included studies, the total number of stroke events was 396 in the experimental group and 456 in the control group. The meta-analysis using a random-effects model demonstrated an RR of 0.76 with a 95% CI of 0.57 to 1.00. This suggests a 24% relative risk reduction in stroke with colchicine use, although the confidence interval reaches the null value, indicating borderline statistical significance (p = 0.05). The heterogeneity among studies was moderate, with an I² value of 50%, suggesting variability in effect estimates across studies.

Effect of Colchicine on All-Cause Mortality 

**Figure 3:**
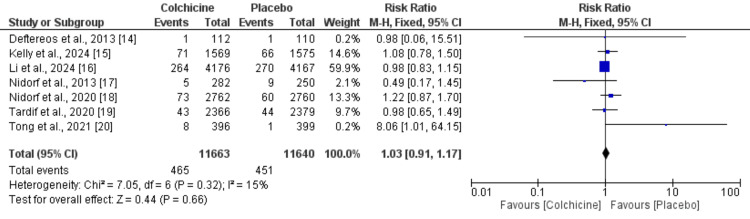
Effect of colchicine on all-cause mortality References [14-20]

The meta-analysis evaluated the effect of colchicine on all-cause mortality, including data from seven studies and the results are presented in Figure 3. The total number of participants was 11,663 in the experimental (colchicine) group and 11,640 in the control group. Across these studies, there were 465 events in the colchicine group and 451 events in the control group. Using a fixed-effects model, the pooled analysis showed an RR of 1.03 with a 95% CI of 0.91 to 1.17. This result suggests no significant difference in all-cause mortality between patients treated with colchicine and those in the control group (p = 0.66). The heterogeneity was low, as indicated by an I² value of 15% suggesting that the variation among the studies was not substantial. 

Discussion 

The findings of this systematic review and meta-analysis suggest that colchicine may have a potential role in stroke prevention, although the results should be interpreted with caution. Our analysis, which included seven studies with a total of 23,303 participants, demonstrated a borderline significant 24% relative risk reduction in stroke incidence among patients treated with colchicine compared to control groups (RR 0.76, 95% CI 0.57-1.00, p = 0.05). This effect is consistent with previous meta-analyses that have reported stroke reduction with colchicine use, particularly in patients with coronary artery disease [13]. 

The magnitude of stroke risk reduction observed in our study is notable, albeit less pronounced than some previous reports. For instance, a meta-analysis by Atta et al. found a 52% relative risk reduction in stroke incidence with colchicine use in coronary artery disease patients [21]. Similarly, another meta-analysis reported a significant reduction in stroke risk with an odds ratio of 0.33 (95% CI 0.15-0.70) [22]. The difference in effect size between our study and these previous analyses may be attributed to our inclusion of more recent trials and our focus on a broader patient population. 

The mechanism by which colchicine may reduce stroke risk is likely multifaceted. Its anti-inflammatory properties are thought to play a crucial role in stabilizing atherosclerotic plaques and reducing vascular inflammation [23]. This effect is particularly important given the growing recognition of inflammation as a key target for reducing residual cardiovascular risk [21]. Additionally, some studies have suggested that colchicine may have beneficial effects on atrial fibrillation, carotid atherosclerosis progression, and aortic aneurysm stability, all of which could contribute to stroke prevention [23]. 

However, it is important to note that not all studies have shown consistent benefits. The recent CHANCE-3 trial, which specifically examined colchicine in patients with acute non-cardioembolic minor-to-moderate ischemic stroke or transient ischemic attack, did not find a significant reduction in subsequent stroke risk within 90 days [16]. This highlights the need for careful consideration of patient populations and treatment timing when evaluating colchicine's efficacy in stroke prevention. 

Our analysis of all-cause mortality showed no significant difference between colchicine and control groups (RR 1.03, 95% CI 0.91-1.17, p = 0.66). This finding is reassuring from a safety perspective and consistent with previous studies that have generally found colchicine to be well-tolerated [21]. However, it also underscores the importance of weighing potential benefits against the lack of mortality reduction when considering colchicine for stroke prevention. The heterogeneity observed in our stroke analysis (I² = 50%) suggests variability in the effect of colchicine across studies. This could be due to differences in patient populations, colchicine dosing regimens, or duration of follow-up. Future research should aim to identify subgroups of patients who may derive the greatest benefit from colchicine therapy. 

In conclusion, while our meta-analysis suggests a potential benefit of colchicine in stroke prevention, the borderline significance of the result calls for cautious interpretation. The findings support the need for larger, well-designed randomized controlled trials specifically focused on stroke outcomes. Such trials should aim to clarify the optimal patient population, dosing regimen, and timing of colchicine initiation for maximum benefit in stroke prevention. As the field of anti-inflammatory therapy in cardiovascular disease continues to evolve, colchicine remains a promising agent worthy of further investigation in the context of comprehensive stroke prevention strategies. 

Study Limitations 

The relatively small number of included studies (seven) limits the statistical power and generalizability of the findings. The moderate heterogeneity observed (I² = 50%) suggests variability in effect sizes across studies, which could be due to differences in patient populations, colchicine dosing regimens, or follow-up durations. The borderline statistical significance (p = 0.05) for stroke reduction indicates uncertainty in the true effect size. The analysis is based on study-level data rather than individual patient data, which limits the ability to explore patient-level factors influencing outcomes. The inclusion of studies with varying primary endpoints and definitions of stroke may introduce some inconsistency. Additionally, the lack of long-term follow-up data in most included studies limits conclusions about the sustained efficacy and safety of colchicine for stroke prevention. Publication bias, though not formally assessed, could potentially influence the results. 

Research Implications 

Based on the findings of this meta-analysis, several important research implications emerge. Larger, well-designed randomized controlled trials specifically focused on stroke outcomes are needed to confirm the efficacy of colchicine in stroke prevention. Future studies should aim to identify optimal patient populations who may benefit most from colchicine therapy and determine the ideal dosing regimen and timing of initiation for maximum benefit. Mechanistic studies are warranted to elucidate the specific pathways by which colchicine reduces stroke risk, beyond its known anti-inflammatory effects. Long-term safety and efficacy data are required to assess the sustained benefits and potential risks of colchicine use for stroke prevention. Additionally, comparative effectiveness studies comparing colchicine to other anti-inflammatory agents in stroke prevention could help guide clinical decision-making and optimize treatment strategies. 

## Conclusions

This systematic review and meta-analysis provides preliminary evidence supporting colchicine's potential in stroke prevention, demonstrating a borderline significant 24% relative risk reduction. While promising, these findings necessitate cautious interpretation due to moderate heterogeneity and borderline statistical significance. The results highlight colchicine's potential anti-inflammatory mechanisms in reducing stroke risk, particularly among patients with atherosclerotic cardiovascular disease.

The study underscores the need for larger, more focused randomized controlled trials to definitively establish colchicine's role in stroke prevention. Future research should prioritize identifying optimal patient populations, determining precise dosing strategies, and exploring long-term efficacy and safety. As the understanding of inflammatory pathways in cardiovascular disease evolves, colchicine remains a compelling candidate for comprehensive stroke prevention strategies, warranting continued investigation and rigorous scientific scrutiny.
